# Decreased CRRT Filter Lifespan in COVID-19 ICU Patients

**DOI:** 10.3390/jcm10091873

**Published:** 2021-04-26

**Authors:** David Legouis, Maria F. Montalbano, Nils Siegenthaler, Camille Thieffry, Benjamin Assouline, Pierre Emmanuel Marti, Sebastian D. Sgardello, Claudio Andreetta, Céline Binvignat, Jérôme Pugin, Claudia Heidegger, Frédéric Sangla

**Affiliations:** 1 Intensive Care Unit, Department of Acute Medicine, Geneva University Hospitals, Rue Gabrielle Perret-Gentil 4, CH-1211 Geneva, Switzerland; david.legouis@hcuge.ch (D.L.); mariaflorencia.montalbano@hcuge.ch (M.F.M.); nils.siegenthaler@hcuge.ch (N.S.); camille.thieffry@hcuge.ch (C.T.); benjamin.assouline@hcuge.ch (B.A.); pierre-emmanuel.marti@hcuge.ch (P.E.M.); sebastiandouglas.sgardello@hcuge.ch (S.D.S.); claudio.andreetta@hcuge.ch (C.A.); celine.binvignat@hcuge.ch (C.B.); jerome.pugin@hcuge.ch (J.P.); Claudia.Heidegger@hcuge.ch (C.H.); 2 Laboratory of Nephrology, Department of Medicine and Cell Physiology and Metabolism, University of Geneva, Rue du Général-Dufour 24, CH-1211 Geneva, Switzerland; 3 University of Geneva, Rue du Général-Dufour 24, CH-1211 Geneva, Switzerland

**Keywords:** COVID-19, continuous renal replacement therapy, filter lifespan, ICU, coagulation, heparin

## Abstract

(1) Background: Increased thromboembolic events and an increased need for continuous renal replacement therapy (CRRT) have been frequently reported in COVID-19 patients. Our aim was to investigate CRRT filter lifespan in intensive care unit (ICU) COVID-19 patients. (2) Methods: We compared CRRT adjusted circuit lifespan in COVID-19 patients admitted for SARS-CoV-2 infection to a control group of patients admitted for septic shock of pulmonary origin other than COVID-19. Both groups underwent at least one session of CRRT for AKI. (3) Results: Twenty-six patients (13 in each group) were included. We analysed 117 CRRT circuits (80 in the COVID-19 group and 37 in the control group). The adjusted filter lifespan was shorter in the COVID-19 group (17 vs. 39 h, *p* < 0.001). This trend persisted after adjustment for confounding factors (−14 h, *p* = 0.037). Before CRRT circuit clotting, the COVID-19 group had a more procoagulant profile despite higher heparin infusion rates. Furthermore, we reported a decreased relation between activated partial thromboplastin time (aPTT) and cumulative heparin dose in COVID-19 patients when compared to historical data of 23,058 patients, suggesting a heparin resistance. (4) Conclusion: COVID-19 patients displayed a shorter CRRT filter lifespan that could be related to a procoagulant profile and heparin resistance.

## 1. Introduction

Acute kidney injury (AKI) is frequently associated with critically ill coronavirus disease (COVID-19) patients and occurs in a third of intensive care unit (ICU) patients, of whom 10 to 20% require continuous renal replacement therapy (CRRT) [1,2,3]. The main limitation of CRRT is the premature clotting of the filters leading to blood loss, decreased effectiveness and a longer duration of treatment which in turn increases costs [4,5]. Optimizing filter life and the performance efficiency of CRRT has been the focus of recent research. Numerous factors pertaining to CRRT modalities, clinical features, biological parameters and coagulation parameters have been identified [6].

Due to their highly inflammatory state, patients with COVID-19 express hypercoagulability [7], and therefore have a higher risk of vascular thromboembolisms [8,9,10,11,12,13]. Consequently, the alteration of coagulation in COVID-19 patients may affect the lifespan of the CRRT circuit due to early clotting of filters [14].

In the context of the ongoing global pandemic, ICUs are under increasing strain, to the point where some risk being quickly overwhelmed. It is therefore necessary to assess CRRT filter lifespan in COVID-19 patients and identify the best CRRT setting in order to optimize treatment and reduce workload.

The aim of this study is to compare CRRT filter lifespan in ICU COVID-19 patients with non-COVID-19 ICU patients admitted for septic shock of a pulmonary origin and to identify the CRRT settings associated with a reduced filter lifespan.

## 2. Materials and Methods

### 2.1. Study Design

We conducted a single-centre retrospective cohort study in the medical-surgical adult ICU of the Geneva University Hospitals (Geneva, Switzerland). The study was approved by the ethics committee for human studies of Geneva, Switzerland (CCER 2020-01974, 20 August 2020, Commission Cantonale d’Ethique de la Recherche).

### 2.2. Patient Selection

Adult patients (>18 years of age) were screened from the database of the ICU department. Medical records were independently reviewed by two ICU physicians. All patients admitted to our ICU for SARS-CoV-2 infection between March 2020 and May 2020 were screened from our database of COVID-19 patients. Among them, patients who underwent at least one session of CRRT for AKI were included in the COVID-19 group. The control group included thirteen consecutive patients (the same number of patients as the COVID-19 group) who were admitted for septic shock of a pulmonary origin other than that of COVID-19 and who underwent at least one session of CRRT for AKI. These patients were screened from our ICU database. In both groups, patients with an ICU length of stay of less than 72 h were excluded.

### 2.3. Data Collection

The following data were extracted from medical and biological records upon ICU admission: demographic characteristics, comorbidities, clinical examination findings as well as laboratory and microbiologic records. Furthermore, biological records, CRRT settings (catheter position, anticoagulation, blood flow rate, pre- and post-dilution rate, and dialysate rate) and circuit lifespan for each CRRT session were also extracted.

Any circuit which was replaced because of filter clotting and circuits electively replaced after 72 h of use (according to manufacturer recommendations) were analysed.

Clotting of the filter circuit was defined as (a) persistent transmembrane pressure greater than 250 mm Hg, (b) presence of visible clots in the circuit or (c) any alarm on the device signalling that the filter had clotted.

Circuits electively discontinued (due to vascular access problems, intra-hospital transport, death or withdrawal of artificial life support) were excluded from the study. CRRT filter lifespan was then defined as the filters’ longevity (in hours) before needing replacement.

As some circuits were used for more than 72 h despite the manufacturer’s recommendations, we included a new variable: ‘adjusted filter lifespan’ which allowed us to set the maximum value of the filter lifespan to 72 h in circuits used for more than 72 h.

In order to investigate the relation between anticoagulation and heparin dose, historical data on activated partial thromboplastin time (aPTT) and the corresponding heparin cumulative dose in all patients admitted to the ICU between January 2010 and September 2020 were extracted from biological records.

### 2.4. CRRT Protocol during Study Period

CRRT was performed using Prismaflex™ devices (Baxter, IL, USA) with the Prismaflex ST150™ filter and the polyacrylinitrile AN69 membrane (Baxter, IL, USA). Femoral or jugular internal veins were used as venous access points with GamCath ^®^ catheters (Gambro, Hechingen, Germany); High Flow; double or triple lumen; 13 Fr; 200 mm, 250 mm for jugular or femoral access respectively. In patients with extracorporeal membrane oxygenation (ECMO), the CRRT circuit was plugged in-line with the ECMO circuit, with blood for CRRT taken from the outlet port (arterial side) and returned to the inlet port (venous side) of the oxygenator membrane.

CRRT was performed in a continuous veno-venous hemodiafiltration therapy mode. Clinicians used local prescription charts based on ideal body weight and a cumulative effluent dose of 30 mL/kg/h and set the blood flow rate between 100–200 mL/min, a dialysis rate of 10 mL/kg/h during heparin anticoagulation and 12 mL/kg/h during citrate anticoagulation and the volume of replacement fluid. Replacement fluid was administered in both pre- and post-dilution with a ratio of either 1/3 pre dilution—2/3 post dilution, or 2/3 pre dilution—1/3 post dilution. In order to improve CRRT efficiency during the COVID-19 period, we initially favoured post dilution (2/3 post dilution). However, after facing frequent filter clotting, pre dilution was then favoured (2/3 pre dilution).

Indications for CRRT initiation were at the discretion of the treating physician.

In cases of increased risk of bleeding or contraindication to citrate anticoagulation, CRRT was performed without any anticoagulation.

When CRRT was performed with anticoagulation therapy, physicians could use unfractionated heparin or regional citrate anticoagulation. Standard practice in our institution is to favour the use of citrate anticoagulation however, during the COVID-19 period, anticoagulation with heparin was used in order to decrease the workload. Furthermore, different dosages of heparin were used to maximize the CRRT filter lifespan. When citrate anticoagulation was prescribed, Regiocit™ citrate enriched solution (Baxter, IL, USA) was used as pre dilution with an initial citrate dose of 3 mmol/kg/h. Citrate dose was adapted to keep postfilter ionized calcium between 0.3 and 0.4 mmol/L.

### 2.5. Objectives

The primary endpoint was the adjusted CRRT circuit lifespan between COVID-19 and the control group. The second aim was to identify CRRT settings and anticoagulation strategies associated with adjusted CRRT filter lifespan.

### 2.6. Statistical Analysis

Continuous variables were expressed as median (25th–75th percentile) and compared using the Mann-Whitney test. Categorical variables were expressed as absolute and relative (%) frequencies, and were compared with Fisher’s exact test. Multivariable analyses were performed using a linear mixed model with a random intercept for each patient and without correlation structure assumption. Results were expressed as an estimate with ±95% confidence intervals. Filter survival data was fitted with a Kaplan-Meier curve and compared using a mixed effects cox model with a random intercept for each patient. The relation between aPTT and heparin infusion rate was first analysed with a linear mixed model with a random intercept for each patient and without correlation structure assumption. We then used simulated heparin infusion rates to plot the predicted relation. A *p-*value of less than 0.05 was considered significant, and all *p-*values were two tailed. Statistical analyses were performed using R software.

## 3. Results

### 3.1. Population

**Figure 1:**
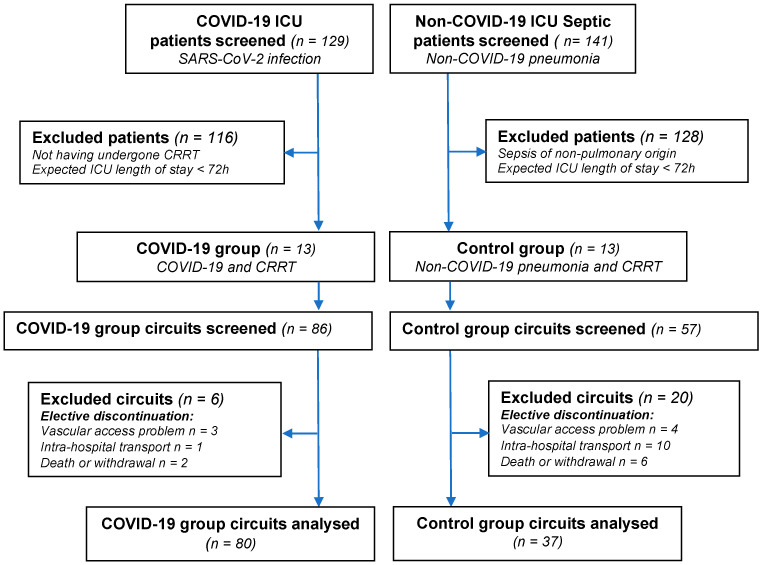
Flow chart of the study.

Among the 129 COVID-19 ICU patients screened, 13 patients requiring CRRT were included in the COVID-19 group (Figure 1). Recruiting 13 patients to form the control group required screening 141 ICU patients admitted for septic shock of all causes between March 2019 and May 2020.

Baseline characteristics of the patients are shown in Table 1. Of the 26 patients included in this study, 143 circuits (86 in the COVID-19 group and 57 in the control group) were screened. Among them, 26 circuits (6 in the COVID-19 group and 20 in the control group) were excluded from the study because of elective discontinuation (Figure 1). Finally, 117 CRRT circuits (80 in the COVID-19 group and 37 in the control group) were analysed.

### 3.2. Filter Lifespan

**Figure 2:**
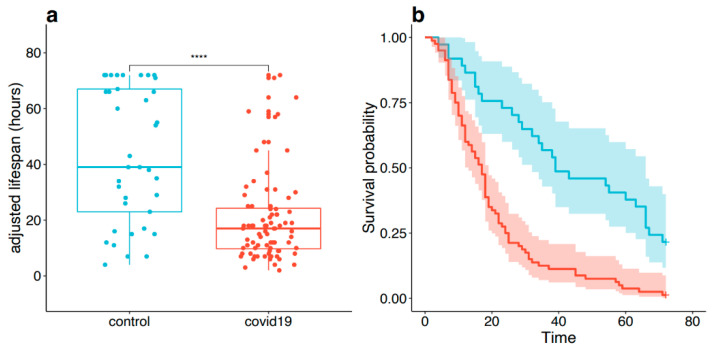
Filter lifespan. (**a**) adjusted filter lifespan in control (blue) and COVID-19 (red) groups shown as boxplot with median and interquartile range. (**b**) survival curves displaying adjusted filter lifespan among COVID-19 (red) and control (blue) groups. **** *p* < 0.0001.

The overall adjusted median of filter lifespan was 19 h (Table 2). It was significantly shorter in the COVID-19 group (17 h vs. 39 h in the COVID-19 and the control group respectively, *p* < 0.001) (Figure 2a). This observation is in line with a higher hazard ratio for filter change observed in the COVID-19 group (HR = 3.26 95%CI [1.6;6.7], *p* = 0.001) (Figure 2b).

In the multivariate analysis, after adjustment for factors identified in the univariate analysis (SAPS II score, platelet count before CRRT circuit initiation, administration of bicarbonate before CRRT circuit initiation, heparin anticoagulation, citrate anticoagulation and diabetes), the COVID-19 group was still associated with a lower filter lifespan (−14 h, *p* = 0.037) (Table 3).

### 3.3. CRRT Settings and Venous Access

CRRT circuit anticoagulation was mostly achieved through the use of heparin in both COVID-19 and the control group (85% and 40.5% respectively). In the control group, 32% of circuits were neither anticoagulated by heparin (risk of bleeding) nor citrate (contraindication to the use of citrate). Replacement fluid was administrated differently in the two groups, with a pre-post dilution strategy ratio of 2/3 pre-1/3 post in all control group patients but in only 72.5% of the COVID-19 patients (Table 2).

Neither dialysate flow rate, pre- or post-dilution strategy nor the type of anticoagulation were significantly related to filter lifespan. On the contrary, femoral venous access was negatively associated with filter lifespan (Table 4).

### 3.4. Anticoagulant Profile

Seeing as the main indication for filter change was filter clotting, we investigated the coagulation profile of patients included in the study. At ICU admission, platelet count, aPTT and fibrinogen rate were not different between groups—whereas the prothrombin time (PT) ratio was higher in the COVID-19 group than in the control group (93% vs. 55% respectively, *p* = 0.043) (Table 1).

**Figure 3:**
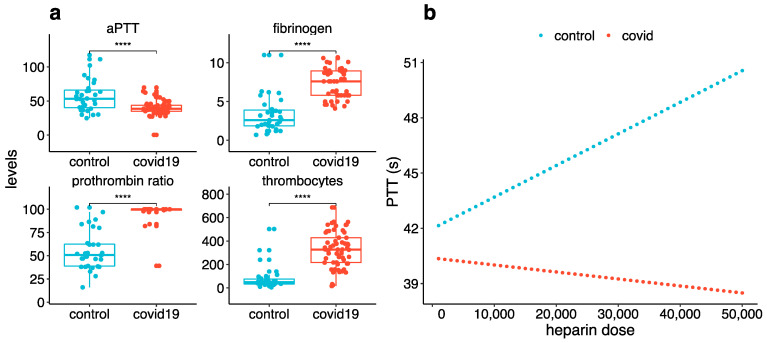
Coagulation profile in COVID-19 (red) and control (blue) groups. (**a**) aPTT level in seconds, fibrinogen level in g/L, prothrombin ration in percentages and thrombocytes level in G/L. (**b**) Simulated aPTT (seconds) according to heparin cumulative dose (UI) in COVID-19 (red) and control (blue) groups. **** *p* < 0.0001.

Before each CRRT circuit initiation and before each CRRT circuit clotting, patients in the COVID-19 group displayed a more procoagulant profile with higher platelet counts, fibrinogen rate and PT ratios as well as a lower aPTT (Table 2 and Figure 3a). Heparin infusion rate was higher in the COVID-19 group than in the control group: median 9.2 (5.8–12.3) UI/kg/h and 4 (3.8–6.5) UI/kg/h respectively, (*p* < 0.001). In order to investigate this apparent discrepancy between heparin infusion rate and coagulation profile, we took advantage of our large historic database including 118,008 aPTT collected in 23,058 ICU patients admitted for all causes between January 2010 and September 2020. We then fitted the relation between heparin dose and aPTT using a linear model including heparin cumulative dose and COVID-19 status as independent variables with an interaction term (Figure 3b). While historical values in 23,058 patients showed an increase in aPTT with higher heparin cumulative dose (1.7 s increase in aPTT for each 10,000 UI of heparin, *p* < 0.001), this relation was not significant in the COVID-19 patient (*p* = 0.4) (Figure 3b).

### 3.5. Kidney Function Recovery

Kidney function recovery was not different between groups (Table 1). Neither filter lifespan (OR 1.08, *p* = 0.13), CRRT duration (OR 0.98, *p* = 0.80) or Covid-19 status (OR 0.60, *p* = 0.62) were associated with the recovery of kidney function.

## 4. Discussion

In this retrospective single-centre cohort study, we investigated the CRRT filter lifespan in COVID-19 ICU patients during CRRT. We found a decrease in filter lifespan in these patients compared to ICU patients admitted for septic shock of pulmonary origin other than COVID-19. This association persists in a multivariable regression adjusted for cofounding factors.

We did not find any significant association between CRRT settings and filter lifespan, however the choice of using a femoral venous access was negatively associated with filter lifespan. 

Our results suggest that the filter lifespan is essentially influenced by the patient’s coagulation profile, which is more procoagulant in COVID-19 patients. Interestingly, heparin effectiveness appeared to be reduced in this patient population.

This difference in filter lifespan is not related to a higher median filter lifespan in the control group.

We reported an adjusted median filter lifespan of 39 (23–67) hours in our control group which is in line with other works in the literature, reporting filter patency ranging from of 37 to 55 h in citrate anticoagulated CRRT, and 26 to 50 h in heparin anticoagulated CRRT [15,16].

The difference in filter lifespan related to a lower median filter lifespan in the COVID-19 group was also described by recently published data from *Endres* et al. who reported a median CRRT first filter lifespan of 6.5 (2.5–33.5) hours in COVID-19 patients [17].

Among CRRT-related factors associated with CRRT filter lifespan, SOFA scores had formerly been shown to be associated with a lower filter lifespan [6,18,], but in our study SOFA scores were lower in the COVID-19 group.

Regarding the CRRT settings which may be related to circuit longevity, the lower filter lifespan in COVID-19 group cannot be explained by differences in the dialysate rate as they do not differ between the groups. It was suggested that CRRT settings such as the blood flow rate or the fluid replacement strategy could influence circuit longevity although this remains controversial [6,19]. Nevertheless, for the COVID-19 patients observed in this study, blood flow rate was greater than 100 mL/min and replacement fluid was infused with a predominant pre-dilution modality.

The choice of venous access site could also be associated with filter lifespan but that remains controversial [6]. Our results show that femoral accesses were associated with a decreased CRRT filter lifespan compared to jugular accesses.

Anticoagulation strategies appear to be a major player in filter clotting prevention [20] especially for regional citrate anticoagulation which has been associated with improved filter lifespan [21]. Our observations show that citrate anticoagulation was not significantly different between groups (15% vs. 27% in the COVID-19 and the control group respectively, *p* = 0.12) but the small number of patients included limits the analysis regarding this issue.

When considering the effect of heparin, more patients in the COVID-19 group received heparin (85% vs. 40% in COVID-19 and control groups respectively, *p* < 0.001) at a higher dose (9.2 vs. 4 IU/kg/h, *p* = 0.02).

Coagulation parameters have been described as being associated with CRRT filter lifespan [6,22,23,24,25,26]. Before CRRT circuit clotting, the COVID-19 group had a more procoagulant profile than the control group with a higher platelet count (327 vs. 48, *p* < 0.001), fibrinogen rate (8.5 vs. 2.6, *p* < 0.001) and PT ratio (100 vs. 51 %, *p* < 0.001), as well as a lower aPTT (39 vs. 53, *p* < 0.001) and an International Normalized Ratio (INR) (1 vs. 1.4 *p* < 0.001). Therefore, we could hypothesize that the increased CRRT circuit clotting observed in COVID-19 patients was due to the coagulopathy frequently reported in viral infections [27,28,29]. This is particularly true in COVID-19 infections as reported in the literature, with an incidence of vascular thromboembolism between 11% and 56% in COVID-19 patients [9,30,31]. The main factors normally recognized as having a procoagulant impact and which are present in COVID-19 patients are thombocytemia and higher aPTT levels [27,29]. An increase of d-dimer values is known to be highly predictive of vascular thromboembolism [30], but in our control group, d-dimer values were not reported in 10/13 patients thus preventing a comparison with the COVID-19 group.

The alteration of coagulation and the limited efficacy of heparin observed in COVID-19 patients suggest some degree of heparin resistance. Heparin resistance has already been described in ICU COVID-19 patients [32]. This syndrome is classically defined as the need for a daily heparin dose of more than 35,000 units/d [32,33]. Although only 4/117 of the analysed patients reached these levels of heparin dosage, we observed a suppression of the relation between heparin dose and aPTT in the COVID-19 group while the control group displayed a positive association, as expected. This is in line with the lower aPTT level measured in COVID-19 patients before filter clotting (39.1 versus 51.4 s, *p* < 0.001) despite a higher heparin dose (4 vs. 9.2 Ui/kg/h *p* < 0.001). In addition, COVID-19 patients in our cohort had high serum fibrinogen levels, what is known to be associated with a decrease in aPTT and the development of heparin resistance [34,35].

Our study has some limitations. First, the study was a single-centre study and included a low number of cases which limits the generalization of results. Nonetheless, our results are in accordance with the literature.

Secondly, this was a retrospective study. Different CRRT settings and anticoagulation strategies were used in both groups. Furthermore, the number of circuits in the COVID-19 group which were anticoagulated by citrate was low (*n* = 12, 15%). We must therefore be cautious when concluding that our results show no difference in filter lifespan between circuits anticoagulated by heparin or citrate. As suggested by other studies, citrate anticoagulation is probably associated with higher CRRT filter lifespans [15,36], even in COVID-19 patients [21]. Further studies using standardized CRRT settings and anticoagulation strategies could help to investigate further factors associated with decreased filter lifespan as observed in COVID-19 patients.

## 5. Conclusions

COVID-19 patients displayed a shorter CRRT filter lifespan which could be related to procoagulant profile and heparin resistance. Further and larger studies are needed to confirm these findings.

## Figures and Tables

**Table 1:** Characteristics of included patients.

	COVID-19 (*n* = 13)	Control (*n* = 13)	Total (*n* = 26)	*p-*Value
Patient Characteristics				
Age (years), median (IQR)	68 (57–73)	57 (55–63)	61 (55–69)	0.150
Sex, male, *n* (%)	12 (92.3)	11 (84.6)	23 (88.5)	1.000
Weight (kg), median (IQR)	88 (81–95)	73 (69–96)	83 (71–96)	0.095
BMI, median (IQR)	28 (27–30)	23 (21–32)	27 (23–32)	0.065
Diabetes, *n* (%)	8 (61.5)	1 (7.7)	9 (34.6)	0.011
HTA, *n* (%)	10 (76.9)	5 (38.5)	15 (57.7)	0.111
SAPS II score, median (IQR)	46 (31–74)	81 (57–93)	64 (41–84)	0.024
APACHE II score, median (IQR)	18 (13–31)	33 (28–39)	28 (17–35)	0.022
SOFA score, median (IQR)	7 (6–8)	11 (11–17)	10 (7–11)	<0.001
Primary CRRT Indication				0.065
Acute kidney injury, *n* (%)	8 (61.5)	5 (38.5)	13 (50)	
Electrolyte/acid-base disturbance, *n* (%)	3 (23.1)	8 (61.5)	11(42.3)	
Fluid overload, *n* (%)	2 (15.4)	0 (0)	2 (7.7)	
At Admission				
Diuresis (mL/24 h), median (IQR)	710 (615–1020)	240 (105–450)	532 (229–808)	0.014
Creatinine (µmol/lL), median (IQR)	116 (104–199)	213 (180–278)	189 (105–274)	0.106
Urea (mmol/L), median (IQR)	8.2 (5.9–13.3)	16.7 (13.2–32.2)	13.3 (7.8–19.7)	0.015
pH, median (IQR)	7.33 (7.30–7.35)	7.38 (7.27–7.40)	7.33 (7.29–7.40)	0.878
Bicarbonate (mmol/L), median (IQR)	22.5 (20.7–25.7)	20.1 (17.6–21.6)	21.6 (18.8–23.2)	0.043
Serum Na+ (mmol/L), median (IQR)	136 (133–142)	138 (134–140)	137 (133–142)	1.000
Serum K+ (mmol/L), median (IQR)	4.1 (3.6–4.3)	3.9 (3.5–4.4)	4.0 (3.5–4.4)	0.918
Lactate (mmol/L), median (IQR)	0.8 (0.7–0.9)	2.6 (1.5–3.4)	1.3 (0.8–2.4)	<0.001
Hemoglobin (g/L), median (IQR)	122 (107–130)	89 (75–130)	110 (87–130)	0.095
White blood cells (G/L), median (IQR)	8.7 (5.5–9.8)	14.4 (9.1–26.2)	9.6 (5.7–15)	0.045
ASAT (U/L), median (IQR)	65 (46–111)	58 (33–86)	62 (39–110)	0.644
Bilirubin (µmol/L), median (IQR)	14 (5–19)	39 (17–145)	19 (10.3–47.2)	0.006
Platelets (G/L), median (IQR)	188 (137–247)	109 (81–183)	163 (108–213)	0.077
Fibrinogen (g/L), median (IQR)	5.4 (5–6.6)	4.2 (2.9–4.9)	4.9 (3.5–6.1)	0.053
aPTT (sec), median (IQR)	39 (35–51)	46 (36–53)	40 (35–53)	0.939
INR, median (IQR)	1.11 (1.03–1.21)	1.31 (1.15–1.79)	1.15 (1.05–1.65)	0.072
Prothombin ratio (%), median (IQR)	93 (70–100)	55 (38–75)	74 (42–100)	0.043
Outcomes in ICU and Hospital				
Adjusted CRRT circuit lifespan (h), median (IQR)	15 (12–24)	40 (26–67)	26 (15–50)	0.006
CRRT total duration (d), median (IQR)	6 (5–8)	3 (3–8)	5.5 (3–8)	0.340
Recovery of kidney function ^1^, *n* (%)	10 (76.9)	11 (84.6)	21 (80.8)	0.618
Vasopressors duration (d), median (IQR)	10 (9–16)	5 (4–11)	9.5 (5–13)	0.025
Mechanical ventilation duration (d), median (IQR)	15 (11–17)	11 (5–14)	13 (10–17)	0.042
Bleeding complication, *n* (%)	7 (54)	11 (85)	18 (69)	0.202
Thromboembolic complication, *n* (%)	2 (15)	3 (23)	5 (19)	1.000
Heparin-indiced thrombocytopenia, *n*	0	0	0	
ICU stay (d), median (IQR)	18 (13–24)	14 (9–18)	15 (11–20)	0.181
Hospital stay (d), median (IQR)	48 (30–52)	20 (17–40)	31 (18–49)	0.102
Day 28 mortality, *n* (%)	3 (23.1)	6 (46.2)	9 (34.6)	0.411
ICU mortality, *n* (%)	3 (23.1)	7 (53.8)	10 (38.5)	0.226
Hospital mortality, *n* (%)	3 (23.1)	7 (53.8)	10 (38.5)	0.226

Data are presented as mean (percentage) or as median (interquartile range). BMI = Body Mass Index, SAPS II = Simplified Acute Physiological Score, APACHE II = Acute Physiology and Chronic Health Evaluation, SOFA = Sepsis-Related Organ Failure Assessment, ASAT = Aspartate Aminotransferase, aPTT = Activated Partial Thromboplastin Time, INR = International Normalized Ratio, CRRT = Continuous Renal Replacement Therapy, d = day, ICU = Intensive Care Unit, ^1^ Recovery of kidney function is defined as the absence of dialysis requirement at ICU discharge.

**Table 2:** Profile of CRRT Circuits.

	COVID-19 (*n* = 80)	Control (*n* = 37)	Total (*n* = 117)	*p*-Value
CRRT circuit characteristics and settings				
Catheter position				<0.001
Femoral, *n* (%)	71 (88.7)	19 (51.4)	90 (76.9)	
Internal jugular, *n* (%)	7 (8.8)	18 (48.6)	25 (21.4)	
ECMO, *n* (%)	2 (2.5)	0 (0)	2 (1.7)	
Anticoagulation				<0.001
Citrate, *n* (%)	12 (15)	10 (27)	22 (18.8)	
Heparin, *n* (%)	68 (85)	15 (40.5)	83 (70.9)	
None, *n* (%)	0 (0)	12 (32.4)	12 (10.3)	
Heparin dose (Ui/kg/h), median (IQR)	9.2 (5.8–12.3)	4 (3.8–6.5)	7.7 (4.7–12)	0.002
Blood flow rate (mL/min), median (IQR)	180 (150–200)	150 (110–200)	180 (150–200)	0.077
Replacement fluid (Pre-Postdilution ratio)				<0.001
1/3–2/3, *n* (%)	22 (27.5)	0 (0)	22 (18.8)	
2/3–1/3, *n* (%)	58 (72.5)	37 (100)	95 (81.2)	
Dialysate rate (mL/h), median (IQR)	800 (800–900)	800 (800–800)	800 (800–900)	0.183
End transmembrane pressure (mmHg), median (IQR)	226 (147–300)	98 (85–188)	200 (93–280)	<0.001
Adjusted CRRT circuit lifespan (h), median (IQR)	17 (10–24)	39 (23–67)	19 (11–38)	<0.001
Before CRRT Circuit Initiation				
Platelets (G/L), median (IQR)	338 (242–451)	54 (33–133)	258 (110–376)	<0.001
Fibrinogen (g/L), median (IQR)	7.6 (5.8–8.9)	2.6 (1.8–3.9)	5.8 (3.2–8.3)	<0.001
aPTT (sec), median (IQR)	39.1 (34.9–46.3)	51.4 (40.2–68.7)	41.5 (35.2–52.2)	<0.001
INR, median (IQR)	1 (1–1)	1.5 (1.3–1.7)	1.1 (1.0–1.5)	<0.001
Prothombin ratio (%), median (IQR)	100 (98–100)	47 (39–63)	80 (45–100)	<0.001
Before CRRT Circuit Clotting				
Platelets (G/L), median (IQR)	327 (216–429)	48 (33–75)	217 (54–372)	<0.001
Fibrinogen (g/L), median (IQR)	8.5 (5.9–9.8)	2.6 (1.9–3.9)	4.1 (2.3–8.3)	<0.001
aPTT (sec), median (IQR)	39 (35–44)	53 (40–66)	41 (35–53)	<0.001
INR, median (IQR)	1 (1–1)	1.4 (1.3–1.8)	1.1 (1–1.5)	<0.001
Prothombin ratio (%), median (IQR)	100 (99–100)	51 (39–62)	81 (48–100)	<0.001

Data are presented as mean (percentage) or as median (interquartile range). CRRT = Continuous Renal Replacement Therapy, ECMO = Extracorporeal Membrane Oxygenation, aPTT = Activated Partial Thromboplastin Time, INR = International Normalized Ratio, ICU = Intensive Care Unit.

**Table 3:** Associated variables with filter lifespan.

	ß	95% CI	*p*-Value
COVID-19 group (vs. control group)	−14.42	−27.30 −1.54	0.037
SAPS II score	0.19	−0.01 0.39	0.079
Platelets before CRRT circuit initiation	−0.03	−0.06 0.002	0.074
Bicarbonate before CRRT circuit initiation	2.47	1.24 3.69	0.0001
Anticoagulation heparin	16.92	4.90 28.94	0.007
Anticoagulation citrate	18.31	5.28 31.33	0.007
Diabetes	−10.80	−21.04 −0.56	0.049

SAPS II = Simplified Acute Physiological Score.

**Table 4:** CRRT setting and filter lifespan in whole cohort.

	ß	95% CI	*p*-Value
Settings			
Dialysate flow rate	0.004	−0.02 0.03	0.788
Replacement fluid ratio 2/3–1/3	−3.31	−13.48 6.86	0.520
Blood flow rate	−0.06	−0.17 0.05	0.280
Anticoagulation			
No anticoagulation	ref		
Heparin < 5	11.53	−2.18 25.23	0.099
Heparin > 5–10	9.608	−5.99 25.21	0.225
Heparin > 10	15.98	−0.33 32.29	0.055
Citrate regional anticoagulation	15.01	−1.95 31.96	0.083
Venous Access			
Internal jugular access		ref	
Femoral access	−12.03	−23.42 −0.64	0.039
ECMO access	4.73	−39.58 49.03	0.834

## Data Availability

Data are available on request from the corresponding author.
